# Quadrivalent human papillomavirus vaccine and autoimmune adverse events: a case–control assessment of the vaccine adverse event reporting system (VAERS) database

**DOI:** 10.1007/s12026-016-8815-9

**Published:** 2016-07-13

**Authors:** David A. Geier, Mark R. Geier

**Affiliations:** Institute of Chronic Illnesses, Inc, 14 Redgate Ct, Silver Spring, MD 20905 USA

**Keywords:** Adverse reaction, Gardasil, RA, Vaccination, SLE

## Abstract

Gardasil is a quadrivalent human papillomavirus (HPV4) vaccine that was approved for use by the US Food and Drug Administration in June 2006. HPV4 vaccine is routinely recommended for administration to women in the USA who are 11–12 years old by the Advisory Committee on Immunization Practices. Previous studies suggest HPV4 vaccine administration was associated with autoimmune diseases. As a consequence, an epidemiological assessment of the vaccine adverse event reporting system database was undertaken for adverse event reports associated with vaccines administered from 2006 to 2014 to 6–39 year-old recipients with a listed US residence and a specified female gender. Cases with the serious autoimmune adverse event (SAAE) outcomes of gastroenteritis (odds ratio (OR) 4.627, 95 % confidence interval (CI) 1.892–12.389), rheumatoid arthritis (OR 5.629, 95 % CI 2.809–12.039), thrombocytopenia (OR 2.178, 95 % CI 1.222–3.885), systemic lupus erythematosus (OR 7.626, 95 % CI 3.385–19.366), vasculitis (OR 3.420, 95 % CI 1.211–10.408), alopecia (OR 8.894, 95 % CI 6.255–12.914), CNS demyelinating conditions (OR 1.585, 95 % CI 1.129–2.213), ovarian damage (OR 14.961, 95 % CI 6.728–39.199), or irritable bowel syndrome (OR 10.021, 95 % CI 3.725–33.749) were significantly more likely than controls to have received HPV4 vaccine (median onset of initial symptoms ranged from 3 to 37 days post-HPV4 vaccination). Cases with the outcome of Guillain–Barre syndrome (OR 0.839, 95 % CI 0.601–1.145) were no more likely than controls to have received HPV4 vaccine. In addition, cases with the known HPV4-related outcome of syncope were significantly more likely than controls to have received HPV4 vaccine (OR 5.342, 95 % CI 4.942–5.777). Cases with the general health outcomes of infection (OR 0.765, 95 % CI 0.428–1.312), conjunctivitis (OR 1.010, 95 % CI 0.480–2.016), diarrhea (OR 0.927, 95 % CI 0.809–1.059), or pneumonia (OR 0.785, 95 % CI 0.481–1.246) were no more likely than controls to have received HPV4 vaccine. Confirmatory epidemiological studies in other databases should be undertaken and long-term clinical consequences of HPV-linked SAAEs should be examined.

## Introduction

Genetic, immunological, hormonal, and environmental factors are considered to be important triggers for autoimmune diseases [1]. The ability of vaccination to induce autoimmune illness has been debated in the literature for decades and is often surrounded by controversy [2]. It was postulated that vaccination may induce autoimmunity using similar mechanisms as occurring in infection-induced autoimmunity [1].

Gardasil (Merck & Co, Inc, Whitehouse Station, NJ, USA) is a quadrivalent human papillomavirus (HPV4) vaccine prepared from the purified virus-like particles (VLPs) of the major capsid (L1) protein of HPV Types 6, 11, 16, and 18 [3]. There are approximately 20 µg of HPV 6 L1 protein, 40 µg of HPV 11 L1 protein, 40 µg of HPV 16 L1 protein, and 20 µg of HPV 18 L1 protein in each 0.5 mL HPV4 vaccine dose. There are also approximately 225 µg of aluminum, 9.56 mg of sodium chloride, 0.78 mg of l-histidine, 50 µg of polysorbate 80, 35 µg of sodium borate, <7 µg yeast protein, and water for injection in each 0.5 mL dose of HPV4 vaccine. A preservative or antibiotics are not present in HPV4 vaccine. HPV4 vaccines are to be administered over 6 months to women between the ages of 9 and 26 years of age based upon its approval by the US Food and Drug Administration (FDA) in June 2006, and the HPV4 vaccine is routinely recommended for administration to women who are 11–12 years old by the Advisory Committee on Immunization Practices (ACIP) [4].

As described in a recent review [5], the routine administration of HPV4 was associated with several cases of onset or exacerbations of autoimmune diseases following immunization in the literature and pharmacovigilance databases, triggering concerns about its safety. In light of the potential for HPV4 vaccine to be associated with autoimmunity, a comprehensive case–control epidemiological study of the vaccine adverse event reporting system (VAERS) database to evaluate the risk for reported serious autoimmune adverse events (SAAEs) following HPV4 vaccination was undertaken. The purpose of the present study was to attempt to confirm and extend previous findings of a significant relationship between HPV4 vaccination and SAAEs.

## Materials and methods

The VAERS is an epidemiological database that has been maintained jointly by the US Centers for Disease Control and Prevention (CDC) and FDA since 1990 as a surveillance tool to evaluate vaccine safety. Specific adverse events following vaccination are required to be reported to this database as mandated by law, but other adverse events that occur following vaccine administration are passively reported to VAERS. The VAERS Working Group of the CDC has previously acknowledged that less than 5 % of the total adverse events reported to VAERS are reported by parents. Specific serious adverse events and deaths reported to VAERS are followed-up by the CDC/FDA. The VAERS Working Group of the CDC and the FDA has repeatedly analyzed and published epidemiologic studies based upon VAERS [6, 7].

The VAERS Working Group notes that VAERS is simple to use, flexible by design, and the data are available in a timely fashion, but it also warns that the potential limitations may include systematic error due to underreporting, erroneous reporting, frequent multiple exposures, multiple outcomes, and lack of precise denominators. In addition, when evaluating data from VAERS, it is important to note that, for any reported event, no cause and effect relationship has been established. VAERS is interested in all potential associations between vaccines and adverse events. Therefore, VAERS collects information on any adverse event following vaccination reported by an individual associating the adverse event with vaccination, be it coincidental or truly caused by a vaccine [6, 7].

### Determining the population at risk

An analysis of the VAERS updated through November 2015 was undertaken using the CDC Wonder online computer interface (http://wonder.cdc.gov/vaers.html) and MedAlerts online computer interface (http://www.medalerts.org/vaersdb/index.php). These portals provide a direct method for independent investigators to rapidly analyze up-to-date data in VAERS. Adverse event reports associated with vaccines administered from January 2006 through December 2014 to recipients between 6 and 39 years old with a listed residence in the USA and a specified female gender was used to identify cases and controls in the present study. Overall, a total of 48,852 adverse event reports in females were examined in the present study, and these adverse event reports were reported to VAERS following administration of HPV4 or any other vaccine(s).

### Determining cases

The SAAE cases were selected from the 48,852 total adverse event reports in females examined in the present study and were defined with outcomes specified as gastroenteritis (VAERS code: 10017888), rheumatoid arthritis (VAERS code: 10039073), Guillain–Barre syndrome (VAERS code: 10018767), thrombocytopenia (VAERS codes: 10043554 [thrombocytopenia] or 10043561 [thrombocytopenic purpura]), systemic lupus erythematosus (VAERS code: 10042945), vasculitis (VAERS code: 10047115), alopecia (VAERS codes: 10001760 [alopecia] or 10001761 [alopecia areata] or 10001766 [alopecia totalis]), Central Nervous System (CNS) demyelinating conditions (VAERS codes: demyelination of the CNS [10012305] or multiple sclerosis [10028245] or transverse myelitis [10028527] or optic neuritis [10030942]), ovarian damage (VAERS codes: 10033119 [ovarian abscess] or 10033122 [ovarian atrophy] or 10033132 [ovarian cyst] or 10033136 [ovarian cyst rupture] or 10033137 [ovarian cystectomy] or 10033157 [ovarian enlargement] or 10033165 [ovarian failure] or 10033270 [ovarian necrosis] or 10036049 [polycystic ovaries]), and irritable bowel syndrome (10023003). In addition, general health outcome cases were selected from the 48,852 total adverse event reports in females examined in the present study and were defined with outcomes specified as infection (VAERS code: 10021789), conjunctivitis (VAERS code: 10010741), diarrhea (VAERS code: 10012735), and pneumonia (VAERS code: 10035664). Table 1 summarizes the total number of cases for each type of outcome examined in VAERS. Finally, in order to test the consistency of VAERS and our epidemiological methods used in VAERS with other epidemiological studies of HPV4 vaccine safety, cases with the known HPV4-related outcome of syncope [8, 9] (VAERS code: 10042772) were selected from the from the 48,852 total adverse event reports in females examined in the present study

**Table 1:** A summary of various types of cases and controls examined in the present study

Outcome examined (VAERS code)	Number
*Serious autoimmune adverse events*	
Gastroenteritis (10017888)	25
Controls	48,827
Rheumatoid arthritis (10039073)	43
Controls	48,809
Guillain–Barre syndrome (10018767)	194
Controls	48,658
Thrombocytopenia (10043554 [thrombocytopenia] or 10043561 [thrombocytopenic purpura])	46
Controls	48,806
Systemic lupus erythematosus (10042945)	48,816
Controls	21,998
Vasculitis (10047115)	18
Controls	48,834
Alopecia (10001760 [alopecia] or 10001761 [alopecia areata] or 10001766 [alopecia totalis])	202
Controls	48,650
CNS demyelinating conditions (demyelination of the CNS [10012305] or multiple sclerosis [10028245] or transverse myelitis [10028527] or optic neuritis [10030942])	152
Controls	48,700
Ovarian damage (10033119 [ovarian abscess] or 10033122 [ovarian atrophy] or 10033132 [ovarian cyst] or 10033136 [ovarian cyst rupture] or 10033137 [ovarian cystectomy] or 10033157 [ovarian enlargement] or 10033165 [ovarian failure] or 10033270 [ovarian necrosis] or 10036049 [polycystic ovaries])	55
Controls	48,797
Irritable bowel syndrome (10023003)	28
Controls	48,824
*General health adverse events*	
Infection (10021789)	73
Controls	48,779
Conjunctivitis (10010741)	41
Controls	48,811
Diarrhea (10012735)	1070
Controls	47,782
Pneumonia (10035664)	98
Controls	48,754
*Known HPV4*-*related adverse events*	
Syncope (10042772)	3194
Controls	45,658

### Determining controls

The controls were selected from the 48,852 total adverse event reports in females examined in the present study. The controls were selected for each type of case outcome examined by including only those adverse event reports that did not include the specific type of case outcome under study. Table 1 summarizes the total number of controls for each type of case outcome examined in VAERS.

### Determining exposure

Exposure was determined in the present study based upon HPV4 vaccine administration (VAERS codes: 1098). It was presumed adverse event reports that included HPV4 vaccine were exposed and adverse event reports that did not include HPV4 vaccine were unexposed.

### Statistical analyses

The Fisher’s exact test contained in the StatsDirect (version 2.8.0) statistical software package was utilized for statistical analyses, and a two-sided *p* value <0.05 was considered to be statistically significant. Odds ratios (ORs), *p* values, and 95 % confidence intervals (CIs) were calculated. The null hypothesis was that there would be no difference in exposure to HPV4 vaccine among cases and controls.

## Results

Table 2 examines among cases with SAAEs and controls, the frequency of exposure to HPV4 vaccine administration in the VAERS database. It was observed that cases with the outcomes of gastroenteritis (OR 4.627, 95 % CI 1.892–12.389, *p* < 0.0005), rheumatoid arthritis (OR 5.629, 95 % CI 2.809–12.039, *p* < 0.001), or thrombocytopenia (OR 2.178, 95 % CI 1.222–3.885, *p* = 0.0102), systemic lupus erythematosus (OR 7.626, 95 % CI 3.385–19.366, *p* < 0.0001), vasculitis (OR 3.420, 95 % CI 1.211–10.408, *p* = 0.0102), alopecia (OR 8.894, 95 % CI 6.255–12.914, *p* < 0.0001), CNS demyelinating conditions (OR 1.585, 95 % CI 1.129–2.213, *p* = 0.0065), ovarian damage (OR 14.961, 95 % CI 6.728–39.199, *p* < 0.0001), or irritable bowel syndrome (OR 10.021, 95 % CI 3.725–33.749, *p* < 0.0001) were significantly more likely than controls to have received HPV4 vaccine. It was observed that cases with the outcome of Guillain–Barre syndrome (OR 0.839, 95 % CI 0.601–1.145, *p* = 0.314) were no more likely than controls to have received HPV4 vaccine.

**Table 2:** A summary of exposure to HPV4 vaccine exposure among SAAE cases and controls

Group examined	Number of cases (%)	Number of controls (%)	Odds ratio (95 % CI)	*p* value^a^
*Gastroenteritis*				
Exposed	17	15,367	*4.627 (1.892*–*12.389)*	<*0.0005*
Unexposed	8	33,460		
*Rheumatoid arthritis*				
Exposed	31	15,353	*5.629 (2.809*–*12.039)*	<*0.0001*
Unexposed	12	33,456		
*Guillain*–*Barre syndrome*				
Exposed	54	15,330	0.839 (0.601–1.145)	0.314
Unexposed	140	33,328		
*Thrombocytopenia*				
Exposed	23	15,361	*2.178 (1.222*–*3.885)*	*0.0102*
Unexposed	23	33,460		
*Systemic lupus erythematosus*				
Exposed	28	15,356	*7.626 (3.385*–*19.366)*	<*0.0001*
Unexposed	8	33,460		
*Vasculitis*				
Exposed	11	15,373	*3.420 (1.211*–*10.408)*	*0.0102*
Unexposed	7	33,461		
*Alopecia*				
Exposed	162	15,222	*8.894 (6.255*–*12.914)*	<*0.0001*
Unexposed	40	33,428		
*CNS demyelinating conditions*				
Exposed	64	15,320	*1.585 (1.129*–*2.213)*	*0.0065*
Unexposed	88	33,380		
*Ovarian damage*				
Exposed	48	15,336	*14.961 (6.728*–*39.199)*	<*0.0001*
Unexposed	7	33,461		
*Irritable bowel syndrome*				
Exposed	23	15,361	*10.021 (3.725*–*33.749)*	<*0.0001*
Unexposed	5	33,463		

Italicized odds ratios and *p* values are statistically significant

^a^The Fisher’s exact test was utilized

Table 3 evaluates among cases with general health outcomes and controls, the frequency of HPV4 vaccine administration in the VAERS database. It was observed that cases with the outcomes of infection (OR 0.765, 95 % CI 0.428–1.312, *p* = 0.378), conjunctivitis (OR 1.010, 95 % CI 0.480–2.016, *p* = 0.999), diarrhea (OR 0.927, 95 % CI 0.809–1.059, *p* = 0.272), or pneumonia (OR 0.785, 95 % CI 0.481–1.246, *p* = 0.328) were no more likely than controls to have received HPV4 vaccine.

**Table 3:** A summary of exposure to HPV4 vaccine exposure among general health outcomes cases and controls

Group examined	Number of cases (%)	Number of controls (%)	Odds ratio (95 % CI)	*p* value^a^
*Infection*				
Exposed	19	15,365	0.765 (0.428–1.312)	0.378
Unexposed	54	33,414		
*Conjunctivitis*				
Exposed	13	15,371	1.010 (0.480–2.016)	0.999
Unexposed	28	33,440		
*Diarrhea*				
Exposed	320	15,064	0.927 (0.809–1.059)	0.272
Unexposed	750	32,718		
*Pneumonia*				
Exposed	26	0.785	0.785 (0.481–1.246)	0.328
Unexposed	72			

^a^The Fisher’s exact test was utilized

For the known HPV4 vaccine-related outcome of syncope, a total of 2871 cases were exposed and 1007 cases were not exposed and 13,197 controls were exposed, and 32,461 controls were not exposed. As a consequence, cases with the outcome of syncope were significantly more likely than controls to have received HPV4 vaccine (OR 5.342, 95 % CI 4.942–5.777, *p* < 0.0001).

Table 4 examines the seriousness and timing of the SAAEs that were significantly associated with HPV4 vaccine administration. It was observed among the SAAEs examined that thrombocytopenia (39.13 %), systemic lupus erythematosus (28.57 %), vasculitis (27.27 %) were associated with the highest percentages of life-threatening outcomes. It was also observed among the SAAEs examined that rheumatoid arthritis (35.48 %), CNS demyelinating conditions (34.38 %), and systemic lupus erythematosus (25.00 %) were associated with highest percentages of permanent disabilities. Finally, the median onset of symptoms for the SAAEs examined revealed that vasculitis was associated with closest median onset of symptoms following HPV4 vaccination (3 days), and rheumatoid arthritis was associated with the longest median onset of symptoms following HPV4 vaccination (37 days).

**Table 4:** A summary of SAAEs associated with HPV4 vaccination

Group examined (*n*)	Life threatening (%)^a^	Permanent disability (%)^a^	Median onset of symptoms^b^
Gastroenteritis (17)	1 (5.88)	3 (17.65)	7.5
Rheumatoid arthritis (31)	3 (9.68)	11 (35.48)	37
Thrombocytopenia (23)	9 (39.13)	3 (13.04)	18
Systemic lupus erythematosus (28)	8 (28.57)	7 (25.00)	23.5
Vasculitis (11)	3 (27.27)	2 (18.18)	3
Alopecia (162)	13 (8.02)	20 (12.35)	25
CNS demyelinating conditions (64)	7 (10.94)	22 (34.38)	23
Ovarian damage (48)	2 (4.17)	5 (10.42)	14
Irritable bowel syndrome (23)	3 (13.04)	3 (13.04)	13

^a^Some outcomes may have more than 1 occurrence in any single event report. If data are grouped by any of these items then the number in the may exceed the total number of unique events and the associated percentage of total unique event reports will exceed 100 % in such cases

^b^Calculated based upon those reports with a specified date of onset of symptoms after vaccination

For the known HPV4 vaccine-related outcome of syncope, it was observed that the median onset of symptoms in syncope adverse events (*n* = 2187) closely associated with HPV4 vaccination (0 days). There were 34 (1.55 %) syncope adverse events considered life threatening, and 57 (2.61 %) were associated with permanent disability.

## Discussion

The present epidemiological study of the VAERS database evaluated the potential relationship between HPV4 vaccine administration and the risk of various types of SAAEs. It was observed that the SAAEs of gastroenteritis, rheumatoid arthritis, thrombocytopenia, systemic lupus erythematosus, vasculitis, alopecia, CNS demyelinating conditions, ovarian damage, and irritable bowel syndrome were associated with HPV4 vaccine administration, whereas the SAAE of Guillain–Barre syndrome was not associated with HPV4 vaccine administration. In addition, it was observed that the general health outcomes of infection, conjunctivitis, diarrhea, and pneumonia were not associated with HPV4 vaccine administration. Finally, it was observed that the previously established HPV4-related outcome of syncope was significantly associated with HPV4 vaccine administration. The importance of these findings is that the present study provides additional epidemiological evidence to support a significant association between HPV4 vaccine administration and specific SAAEs.

The results obtained in the present study are consistent with a previous assessment of the VAERS database for the relationship between HPV4 vaccine administration and SAAEs [10]. The previous study of VAERS revealed a significant link between HPV4 vaccine administration and the outcomes of gastroenteritis, arthritis, systemic lupus erythematosus, vasculitis, alopecia, and CNS conditions [10]. The present assessment of the VAERS is differentiated from the previous assessment of the VAERS because the data examined in the present study included a more updated VAERS database (the previous study examined vaccines administered from January 2006 through December 2012 vs. the present study examined vaccines administered from January 2006 through December 2014) and wider age range of reports (the previous study examined vaccines administered to recipients from 18 to 39 years old vs. the present study examined vaccines administered recipients from 6 to 39 years old).

The results obtained in the present study are consistent with several previous clinical studies. For example, researchers investigated the association between HPV vaccination and autoimmune manifestations compatible with systemic lupus erythematosus or systemic lupus erythematosus-like disease in six women who presented with such symptoms following HPV vaccination [11]. These investigators reported that in their cases, several common features were observed, such as personal or familial susceptibility to autoimmunity or adverse response to a prior dose of the vaccine, both of which may be associated with a higher risk of postvaccination autoimmunity. Favorable response to immunosuppressant was observed in all patients, and there was a temporal association between HPV vaccine and the appearance of systemic lupus erythematosus-like conditions. Similarly, other investigators observed a case series of three women that had onset or exacerbation of lupus following HPV immunization [12]. Other researchers have suggested that an increasing number of cases of ovarian damage has been observed following HPV4 vaccine administration [13].

The results found in the present study are also supported by biological plausible mechanisms of the constitute components of HPV4 vaccine to induce SAAEs. The HPV4 vaccine contains purified VLPs of HPV Types 6, 11, 16, and 18, which are adsorbed on preformed aluminum-containing adjuvant (amorphous aluminum hydroxyphosphate sulfate). The HPV4 vaccine is then prepared by combining the adsorbed VLPs of each HPV type and additional amounts of the aluminum-containing adjuvant in a sterile liquid suspension [3].

A previous female animal model study revealed HPV4 vaccine administration via its aluminum-containing adjuvant, and HPV antigens have the ability to trigger neuroinflammation and autoimmune reactions, further leading to behavioral changes [14]. Other investigators described animal models that showed aluminum exposure to inhibit expression of female reproductive hormones and to induce histologic changes in the ovaries [13].

In contrast to the results observed in the present study and observed by other investigators, Arnheim-Dahlstrom et al. [15] evaluated autoimmune, neurological, and venous thromboembolic adverse events after immunization of adolescent girls with HPV4 in Denmark and Sweden. These investigators examined a cohort of 997,585 girls aged 10–17, among whom 296,826 received a total of 696,420 HPV4 vaccine doses. Unlike the present study that examined adverse event reports to the VAERS database, Arnheim-Dahlstrom et al. examined incident hospital diagnosed autoimmune, neurological, and venous thromboembolic events (53 different outcomes) up to 180 days after each HPV4 vaccine dose, and rate ratios of the outcomes were adjusted for age, country, calendar year, parental country of birth, education, and socioeconomic status, comparing vaccinated and unvaccinated person-time. These investigators observed that the rate ratios for 20 of 23 autoimmune events were not significantly increased. Exposure to HPV4 vaccine was significantly associated with Behcet’s syndrome, Raynaud’s disease, and Type 1 diabetes, but these investigators described that each of these three outcomes fulfilled only one of three predefined signal strengthening criteria. In addition, these investigators observed that the rate ratios for five neurological events were not significantly increased and there was no association between exposure to HPV4 vaccine and venous thromboembolism. It is interesting to note that despite the number of individuals examined by Arnheim-Dahlstrom et al., their study was significantly underpowered to even examine many types of outcomes, and of those outcomes examined, in many cases, relatively few outcomes were observed in the HPV4 vaccinated group. This may potentially be a consequence of the fact that the source for detecting outcomes in the Arnheim-Dahlstrom et al. study was from hospital records. It seems reasonable to hypothesize that many of the conditions examined by Arnheim-Dahlstrom et al. would not necessarily require hospitalization, and worse still, even if the condition eventually might require hospitalization, the Arnheim-Dahlstrom et al. study examined the diagnosis of the outcome in a hospital within the first 181 days after vaccination. As a result, for many of the outcomes examined by Arnheim-Dahlstrom et al. that overlap that with the outcomes examined in the present study, it was observed that there similar potential trends for outcomes in both studies (i.e., odds ratio for vasculitis in VAERS = 3.420 versus adjusted rate ratio in Arnheim-Dahlstrom study = 1.55 or odds for systemic lupus erythematosus in VAERS = 7.626 versus adjusted rate ratio in Arnheim-Dahlstrom study = 1.35), but none of the outcomes were significantly associated with HPV4 vaccination in the Arnheim-Dahlstrom et al. study.

Also in contrast to the present study findings, Chao et al. [16] undertook an observation safety study of HPV4 in 189,629 women who receive one or more doses of HPV4 vaccine between August 2006 and March 2008 for new diagnoses of autoimmune conditions within 180 days following each dose of HPV vaccine. These investigators identified new-onset autoimmune conditions among HPV recipients by electronic medical records, but then the medical records were reviewed by clinicians to confirm the diagnosis and determine the date of onset (only 31–40 % of the cases were confirmed as new onset). It was observed that there was no cluster of disease onset in relations to vaccination timing, dose sequence or age was found for any autoimmune condition. None of the incidence rate ratios was significantly elevated except for Hashimoto’s disease, but further investigation failed to reveal consistent evidence for a safety signal for autoimmune thyroid conditions. These investigators concluded that no autoimmune safety signal was found in women vaccine with HPV4. Once again, the Chao et al. [16] study, just like the previously discussed Arnheim-Dahlstrom et al. [15] study, was apparently significantly underpowered to find potential autoimmune conditions associated with HPV vaccination, since more than half of the new-onset autoimmune cases identified from electronic medical records were subsequently eliminated from the study following review by clinicians.

Another study by Grimaldi-Bensouda et al. [17] evaluated whether HPV4 vaccination was associated with newly diagnosis autoimmune conditions. In Grimaldi-Bensouda et al. study a total of 211 female cases aged 14–26 years old from across France and diagnosed with autoimmune diseases, and 875 controls were recruited from general practices and were examined. There were no significant increases in the risks of diagnosed thrombocytopenia, multiple sclerosis, connective disorders, Type 1 diabetes, Guillain–Barre syndrome or thyroiditis. The investigators concluded that none of the autoimmune disorders studied had an increased risk following HPV4 vaccination within the time periods studied, but the authors acknowledged that their study was limited by statistical power. Yet again, the Grimaldi-Bensouda et al. study, just like the previously discussed Chao et al. [16] and Amheim-Dahlstrom et al. [15] studies, was significantly underpowered to find potential autoimmune conditions associated with HPV vaccination, since there were fewer than 10 cases exposed for each of the autoimmune disorders examined (thrombocytopenia = 6, connective tissue disorders = 6, central demyelination = 4, Guillain–Barre syndrome = 0, and Type 1 diabetes = 9).

### Strengths/limitations

It was previously described that an “unmasking” phenomenon is a concern with many epidemiological studies evaluating vaccine safety among adolescents and young adults because they may undergo a workup and subsequent diagnosis of preexisting conditions following a visit at which a vaccination is administered [18]. The epidemiological method employed to examine VAERS database in this study ensured that the exposures to the various types of vaccines studied occurred prior to the outcomes described in the adverse event reports, since those reporting the adverse outcomes associated the outcomes with the vaccines listed in the adverse event reports. Further, since everyone examined in the present study had to have received a vaccine (i.e., vaccine receipt is a necessary requirement for reporting to VAERS), it is believed that this “unmasking” phenomenon should have applied equally to everyone examined.

Another strength was that the VAERS data were collected independently of the study design used in the present study. Among those reporting the adverse event reports examined, it was highly unlikely that any of them could have envisioned methods of analysis used to evaluate the potential relationship between HPV4 vaccine and the adverse events examined.

An additional strength of the present study was the specificity of the types of the SAAEs associated with HPV4 vaccine. Namely, despite the fact that a number of different types of potential SAAEs were examined for the relationship with HPV4 vaccine administration, it was observed that the types of SAAE outcomes significantly associated with HPV4 vaccination are biologically plausibly associated with HPV4 vaccine administration from previous clinical and animal model studies. Further, previous epidemiological assessment of the VAERS database revealed that laboratory findings consistent with the SAAE outcomes found to be significantly linked with HPV4 vaccine administration in the present study, such as positive rheumatoid factor, positive antinuclear antibodies, and positive antiphospholipid antibodies were significantly associated with HPV4 vaccine administration [10]. Finally, among the SAAEs associated with HPV4 vaccine administration, it was observed, consistent with the biological plausible onset window for SAAEs following vaccination, that the median onset of symptoms ranged between 3 and 37 days post-immunization.

However, the results observed in this study may have a number of potential limitations. It is possible the results observed may have occurred from unknown biases or cofounders present in the datasets examined. This seems unlikely because the general health outcomes of infection, conjunctivitis, diarrhea, and pneumonia were examined, and all were observed to be exposed to HPV4 vaccine at a similar frequency as controls. In addition, the known HPV4-related outcome of syncope was examined in the present study, and it was observed that the risk of syncope (OR 5.342, 95 % CI 4.942–5.777) was very similar to that reported by previous investigators examining California Kaiser Permanente medical records (OR 6.0, 95 % CI 3.9–9.2) [9].

An additional potential limitation of this study is that VAERS may have shortcomings, such as underreporting, difficulty in determining causal relationship, and a lack of precise denominators. Nevertheless, as previously described by investigators from the CDC, almost all of these types of shortcomings would apply equally to VAERS reports after vaccines administered to similar populations [19]. The case–control method employed in the present study ensured that all of the adverse event reports examined in VAERS were administered to similar populations (i.e., age and gender), and as a result, examining the relative exposure to HPV4 vaccine among the cases and controls identified from the adverse event reports examined in VAERS should provide accurate relative qualitative and quantitative relationships between differing vaccine exposures and adverse outcomes. Additionally, investigators previously employed similar case–control study designs to the one used in the present study to successfully evaluate the potential relationship between vaccine administration and various SAAEs [10, 20].

Another potential limitation of the present study is that the results observed may be the result of statistical chance. However, such a possibility would be unlikely given the limited number of statistical tests performed, the highly significant results observed (most *p* values observed were <0.01), and the consistency in the direction and magnitude of the results observed.

Still, other potential limitations of this study include the possibilities that some of the individuals in VAERS may have had more subtle adverse events that were not brought to the attention of their healthcare providers, healthcare providers may have misdiagnosed some individuals, or some vaccine exposures may not have been appropriately classified. These limitations, while possibly present in the data examined in the current study, should not have significantly impacted the results observed because it is unclear how differential application would have occurred based upon different exposures among cases and controls. Moreover, misclassification occurring in the data examined would tend to bias any results observed toward the null hypothesis, since such effects would result in individuals being placed in the wrong exposure and/or outcome categories examined, and result in decreased statistical power to determine true potential exposure-outcome relationships.

In addition, another potential limitation of this study is that other sources of exposure among cases and controls were not evaluated. It is possible that the findings may be the result of other components of the vaccines studied which, in isolation or synergistically, interacted with the HPV4 vaccine examined.

Finally, the current study suffers from the potential limitation that analyses were not conducted to further explore the cumulative dosing effects from HPV4 vaccine administration or to compare HPV vaccine administration with unvaccinated populations. In future studies, it would be worthwhile to further explore these precise-timing and cumulative-doses phenomena. In addition, it would be valuable to evaluate other adverse events, as well as other covariates such as race, prior medical history, etc., that may further affect the magnitude of the adverse effects found.

## Conclusion

In conclusion, this epidemiological study provides additional evidence to support a significant relationship between HPV4 vaccine administration and SAAEs. The results in this study are consistent with a number of previous case series of SAAEs observed following HPV4 vaccine administration, previous epidemiological studies linking HPV4 vaccine with SAAEs and other outcomes, and are consistent with the known biological plausibility of HPV4 vaccine administration to induce SAAEs in some vaccine recipients. In light of the findings of the present study, we recommend that additional studies be conducted to further evaluate the potential epidemiological relationship between HPV4 vaccine-associated SAAEs in other databases and populations and to further evaluate the long-term clinical consequences of HPV-linked SAAEs.
